# EST analysis of male accessory glands from *Heliconius *butterflies with divergent mating systems

**DOI:** 10.1186/1471-2164-9-592

**Published:** 2008-12-08

**Authors:** James R Walters, Richard G Harrison

**Affiliations:** 1 Department of Ecology and Evolution, Cornell University, Ithaca, NY, USA

## Abstract

**Background:**

*Heliconius *butterflies possess a remarkable diversity of phenotypes, physiologies, and behaviors that has long distinguished this genus as a focal taxon in ecological and evolutionary research. Recently *Heliconius *has also emerged as a model system for using genomic methods to investigate the causes and consequences of biological diversity. One notable aspect of *Heliconius *diversity is a dichotomy in mating systems which provides an unusual opportunity to investigate the relationship between sexual selection and the evolution of reproductive proteins. As a first step in pursuing this research, we report the generation and analysis of expressed sequence tags (ESTs) from the male accessory gland of *H. erato *and *H. melpomene*, species representative of the two mating systems present in the genus *Heliconius*.

**Results:**

We successfully sequenced 933 ESTs clustering into 371 unigenes from *H. erato *and 1033 ESTs clustering into 340 unigenes from *H. melpomene*. Results from the two species were very similar. Approximately a third of the unigenes showed no significant BLAST similarity (E-value <10^-5^) to sequences in GenBank's non-redundant databases, indicating that a large proportion of novel genes are expressed in *Heliconius *male accessory glands. In both species only a third of accessory gland unigenes were also found among genes expressed in wing tissue. About 25% of unigenes from both species encoded secreted proteins. This includes three groups of highly abundant unigenes encoding repetitive proteins considered to be candidate seminal fluid proteins; proteins encoded by one of these groups were detected in *H. erato *spermatophores.

**Conclusion:**

This collection of ESTs will serve as the foundation for the future identification and evolutionary analysis of male reproductive proteins in *Heliconius *butterflies. These data also represent a significant advance in the rapidly growing collection of genomic resources available in *Heliconius *butterflies. As such, they substantially enhance this taxon as a model system for investigating questions of ecological, phenotypic, and genomic diversity.

## Background

One of the most promising and productive research approaches in contemporary biology involves deploying modern genomic methods to investigate the origin, maintenance, and function of biological diversity present in natural populations. Research efforts in this nascent field of evolutionary and ecological functional genomics (EEFG) generally can be split into two categories [1,2]. One approach studies natural populations of the few taxa (or their close relatives) that are already well-established laboratory model systems, making use of the extensive molecular genetic and genomic resources available for such organisms (e.g. *Drosophila *and *Arabidopsis*) [3,4]. The alternative approach focuses on taxa which may be less tractable from a methodological perspective but which offer superb opportunities to investigate interesting and important ecological and evolutionary phenomena. In the case of such emerging model taxa, the development of genomic resources such as genetic libraries, linkage maps, and sequence databases are necessary and fundamental first steps in any EEFG research program [2].

*Heliconius *butterflies stand out among emerging model taxa for their extensive history in ecological and evolutionary research [5-12]. The genus *Heliconius*, comprised of ~40 neotropical species, contains a remarkable diversity of phenotypes, behaviors, and physiologies, all of which have evolved relatively recently [7,13-15]. The most conspicuous and well-studied aspect of this diversity is the variation, convergence, and mimicry of wing-color patterns present both within and between species [6,9,16-21]. Efforts to identify the genetic basis of this wing pattern diversity have driven the recent development of genomic resources for *Heliconius *butterflies [20,22,23]. The accumulation of such resources now provides a strong precedent for investigating additional aspects of *Heliconius *diversity.

Here we present the first genomic foray into facets of *Heliconius *diversity other than wing pattern. We focus on the striking dichotomy of mating systems found within the genus and sample the transcriptome of male reproductive tissues from species representative of the two mating systems. Our two focal species are the co-mimetic *H. erato *and *H. melpomene*. These species have an average synonymous site divergence of 14.5%, do not interbreed, show extensive parallel radiations of wing patterns, and are the primary focus for wing pattern research in the genus [20,23,24]. Consequently, these species possess the vast majority of genomic resources available for *Heliconius*: BAC libraries, linkage maps, and extensive collections of expressed sequence tags (ESTs) generated from wing tissue and curated in a lepidopteran-specific database, [20,23,25-27].

*Heliconius erato *and *H. melpomene *represent the two divergent mating systems found in the genus. About half of *Heliconius *species, including *H. erato*, exhibit an unusual pupal mating behavior: females are mated before or during eclosion and typically mate only once (i.e. females are monandrous). Otherwise, *Heliconius *butterflies, such as *H. melpomene*, mate as fully developed adults and regularly mate more than once (i.e. females are polyandrous) [7,8,10,13,28]. *Heliconius *species fall evenly into two major clades which correspond exactly with mating system [13].

This difference in mating system can engender very different regimes of sexual selection between the two clades. For instance, the pupal mating system drives extremely intense pre-mating sexual selection; males compete vigorously for mating position on the female chrysalis [8]. In contrast, the lack of remating in pupal mating females likely precludes most aspects of post-mating sexual selection such as sperm competition and cryptic female choice (reviewed in [29]). Therefore this phylogenetically concordant split in mating systems presents an unusual opportunity to explore hypotheses relating sexual selection to the molecular evolution of reproductive proteins.

Reproductive proteins include proteins mediating gametic interactions or those found in seminal fluid. These proteins tend to diverge rapidly between related species and often evolve via positive Darwinian selection (reviewed in [30-32]). This is a pattern widely observed among animals and also often in plants. It is commonly hypothesized that post-mating sexual selection is the primary evolutionary process underlying this pattern [33-38]. However, there are very few data currently available that directly address this hypothesis (but see [39,40]).

Ultimately we will use the dichotomous mating systems in *Heliconius *to test for a relationship between intensity of post-mating sexual selection and evolutionary rates of reproductive proteins. To do this it is first necessary to identify reproductive proteins in *Heliconius *butterflies. Here again we take our cues from previous EEFG research, though this time not from *Heliconius *but from *Drosophila *fruit flies and from two genera of crickets. In these taxa researchers focused on proteins secreted by the accessory glands – part of the male reproductive tract – into seminal fluid. Early work in this field focused on indirect criteria such as the presence of a signal peptide and accessory gland biased expression to identify genes encoding accessory gland proteins (ACPs) which were assumed to be transferred to females in seminal fluid. Using modest numbers of ESTs generated from cDNA libraries enriched for male-biased transcripts, this work identified dozens of ACPs in these species [36,41,42]. Subsequent studies at the protein level verified that many of these ACPs are transferred to females in seminal fluid [43-47]. These proteins have diverse and often dramatic effects on female reproductive physiology and behavior, including stimulating egg-laying, facilitating sperm storage, and inducing refractoriness to remating [48,49]. Moreover, in *Drosophila melanogaster*, genetic variation in seminal fluid proteins has been correlated with sperm competitive ability, indicating an important link between reproductive protein evolution and sexual selection [50,51].

In this paper we present parallel analyses of ESTs generated from male accessory glands of the pupal mating *H. erato *and the adult mating *H. melpomene*. These data constitute an important first step toward identifying a set of seminal fluid proteins in *Heliconius *butterflies and using these genes to examine the relationship between post-mating sexual selection and the molecular evolution of reproductive proteins. These data also contribute significantly to the development of *Heliconius *butterflies into a sophisticated model system for genomic explorations of ecological and evolutionary phenomena.

## Results and discussion

### Library construction and EST assembly

The accessory glands from 11 adult male *H. erato *and 10 adult male *H. melpomene *were dissected from live butterflies and pooled within species to generate two tissue-specific directional cDNA libraries. The *H. erato *and *H. melpomene *libraries respectively contained 7 × 10^6 ^and 1.3 × 10^5 ^colony-forming units. To enrich for transcripts expressed primarily in male tissue, both libraries were screened with cDNA generated from conspecific female abdominal tissue, and only non-hybridizing clones were sequenced. Approximately 1150 clones were sequenced from each species to generate a collection of ESTs which then were trimmed of low quality reads and poly-A tails, clustered, and assembled into contigs. We presume these assembled clusters, or unique gene objects (*unigenes*), represent distinct transcripts. Results were very similar in both species. *H. erato *yielded 371 unigenes and *H. melpomene *yielded 340. The two libraries were comparable in number of high quality ESTs, average read length, and the frequency spectrum of ESTs per unigene (Tables 1, 2). In both libraries the vast majority of unigenes were represented by a single EST and ~90% of unigenes corresponded to three or fewer ESTs (Table 2).

**Table 1:** Summary of EST, BLAST, and SignalP analyses from *H. erato *and *H. melpomene *male accessory gland cDNA libraries.

	*Heliconius erato*	*Heliconius melpomene*
**EST results**		
Number of clones sequenced	1152	1148
High Quality ESTs^1^	936 (81%)	1033 (89%)
Number of unigenes	371	340
Average sequence length (base pairs)	641	597

**BLAST results^2^**		
Unigenes with significant BLAST hits to GenBank protein or nucleotides^3 ^(E-value < 10^-5^)	257 (69%)	235 (69%)
Unigenes with significant BLASTX hits to GenBank proteins (E-value < 10^-5^)	216 (58%)	218 (64%)
Unigenes with significant BLASTN hits to GenBank nucleotides (E-value < 10^-5^)	151 (40%)	150 (44%)

**SignalP results^2^**		
Unigenes with predicted signal peptides^4^	86 (24%)	92 (28%)

^1^ESTs > 100 bp after trimming raw sequences of vector sequence, low-scoring base calls, and poly-A tails. ^2^Percentages given in reference to total number of unigenes. ^3^Includes results from both BLASTN and BLASTX searches. ^4^We required a positive result from both hidden markov models and neural network methods implemented in SignalP.

**Table 2:** Frequency distribution of ESTs per unigene from *H. erato *and *H. melpomene *male accessory gland cDNA libraries.

ESTs per unigene	*H. erato*	*H. melpomene*
1 (singletons)	319	256
2	13	31
3	5	12
4	9	10
5–10	14	17
11–20	7	9
21–50	3	3
>51	1	2

Total:	371	340

All EST sequences have been submitted to GenBank. *H. erato*, [GenBank: GE841215–GE842150; *H. melpomene*, [GenBank: GE842151–GE843183]. Complete unigene sequences from *H. erato *and *H. melpomene *are available in additional files 1 and 2, respectively.

### Unigene Annotation

We annotated the unigenes using BLASTX and BLASTN to search for similar sequences in GenBank's protein and nucleotide non-redundant databases; a significance cut-off of E-value < 10^-5 ^was used for both searches. Results are summarized in Table 1; the top-five best BLAST hits for each unigene are reported in additional file 3.

Overall, 69% of unigenes in each species yielded significant BLAST hits to GenBank sequences (*H. erato*: 257 of 371 unigenes; *H. melpomene*: 235 of 340). This suggests nearly a third of the unigenes obtained from each species may correspond to novel and previously undescribed genes. In both species many unigenes with significant BLASTN hits to GenBank lacked significant BLASTX hits (*H. erato*: 41 unigenes, 11%; *H. melpomene*: 17 unigenes, 5%). These discrepancies could be explained in one of two ways: 1) these unigenes corresponded to ribosomal RNA or 2) these unigenes contained a *Heliconius *specific novel repetitive element and were similar only to a few other *Heliconius *sequences in GenBank containing such repeats [52] (see section below: *Novel Heliconius repetitive elements*; unigene sequences were not masked for GenBank BLASTs). Nineteen unigene pairs were reciprocal best-BLAST-hits between *H. erato *and *H. melpomene *and also showed no significant similarity to sequences in GenBank.

We used SignalP to identify protein-coding unigenes containing a predicted signal peptide sequence [53]. ACPs are extracellularly secreted proteins and are therefore expected to have a signal peptide [41,42]. Results were again similar between libraries, with 86 (24.4%) secreted proteins in *H. erato *and 92 (27.8%) in *H. melpomene *(Table 1).

### Gene Ontology

Where possible, we assigned Gene Ontology (GO) annotations to protein-coding unigenes using the Annot8r application in the PartiGene software package [54,55]. Annot8r assigns GO terms to unigenes based on BLASTX similarity (E-value < 10^-5^) to proteins with known GO annotations; results are summarized via GO-slim terms corresponding to broad functional classes. GO annotations fall into three independent categories (Biological Process, Molecular Function, and Cellular Component) and a single sequence may be annotated in any or all categories. Moreover, a single sequence may be associated with multiple GO annotations within a single category, giving rise to more GO-annotations than sequences annotated (Table 3). The complete set of GO annotations is provided in additional file 4.

**Table 3:** Counts of GO-slim annotations from *H. erato *and *H. melpomene *accessory gland unigenes broken down by ontological category.

	*H. erato*		*H. melpomene*	
		
	GO-slim Annotations	Unigenes Annotated^1^	GO-slim Annotations	Unigenes Annotated^1^
		
Molecular Function	225	169 (46%)	243	183 (54%)
				
Biological Process	157	126 (34%)	177	151 (44%)
				
Cellular Component	84	82 (23%)	122	121 (36%)
		
Total	466	187 (50%)	542	203 (60%)

Unigenes could be assigned more than one annotation both within and between categories.

^1 ^Percentages given in reference to total number of unigenes.

**Figure 1:**
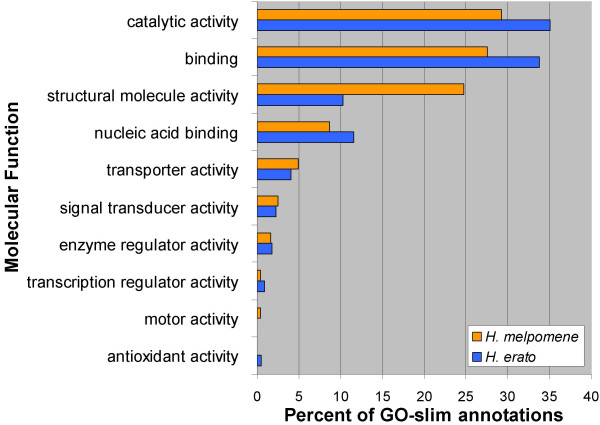
**Molecular function GO-slim annotations from *H. melpomene *and *H. erato *accessory gland unigenes**. Percentages are in reference to total molecular function GO-slim annotations. Not all unigenes could be annotated and some received multiple annotations.

**Figure 2:**
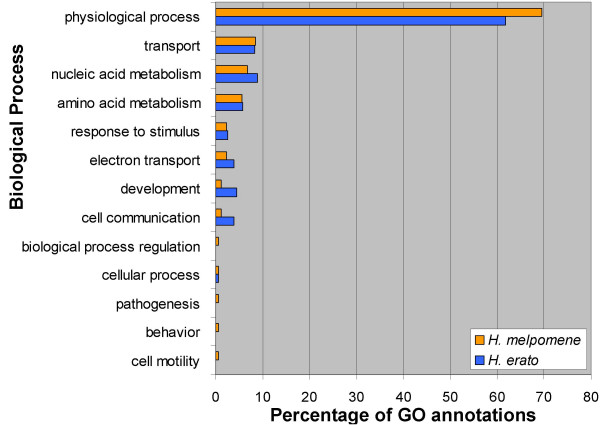
**Biological process GO-slim annotations from *H. melpomene *and *H. erato *accessory gland unigenes**. Percentages are in reference to total biological process GO-slim annotations. Not all unigenes could be annotated and some received multiple annotations.

**Figure 3:**
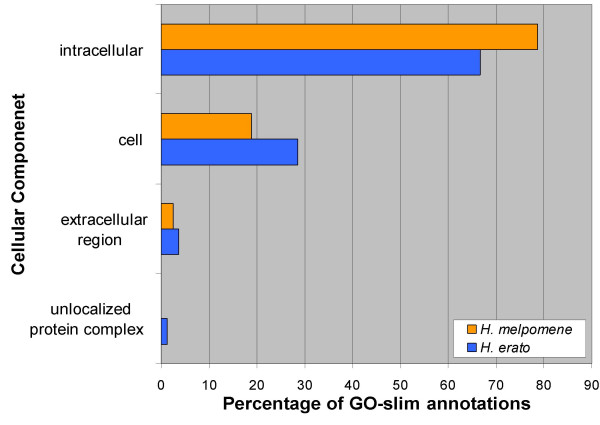
**Cellular component GO-slim annotations from *H. melpomene *and *H. erato *accessory gland unigenes**. Percentages are in reference to total cellular component GO-slim annotations. Not all unigenes could be annotated and some received multiple annotations.

Overall we assigned GO annotations to 187 (50%) and 203 (60%) unigenes from *H. erato *and *H. melpomene*, respectively. With one exception, the distribution of annotations across GO-slim summary terms is quite similar between the two species for all three GO categories (Figs. 1, 2, 3). The one exception is the class "structural molecule activity" in the category Molecular Function (Fig. 1). The proportion of *H. melpomene *annotations in this class is twice that obtained from *H. erato*. This discrepancy clearly results from the greater number of ribosomal proteins represented in the *H. melpomene *ESTs, although it is unclear whether this reflects any biologically significant difference between the two species.

### Novel *Heliconius *repetitive elements

Recently, Papa *et al*. identified nine novel, short (200–600 bp) *Heliconius *specific repetitive elements in BAC sequences from *H. melpomene *and *H. erato *[52]. We used RepeatMasker to identify and mask these repetitive elements in both accessory gland and wing unigenes [56]. Overall, each of the nine repeats were identified among the unigenes, but not all were present in each library (Table 4; Additional file 5).

**Table 4:** Counts of repetitive elements masked among unigenes from *H. erato *and *H. melpomene *accessory glands and developing wing tissue libraries.

	*Heliconius *Repetitive Elements								
	
	**1**	**2**	**3**	**4**	**5**	**6**	**7**	**8**	**9**
***H. erato***									
Accessory Glands	0	2	1	3	0	0	26	3	0
Wing	5	10	19	13	3	5	275	40	1
	
*total*	5	12	20	16	3	5	301	43	1
									
***H. melpomene***									
Accessory Glands	0	0	1	1	0	1	8	1	0
Wing	0	4	0	2	1	1	41	6	1
	
Total	0	4	1	3	1	1	49	7	1

Repetitive element labeling numbers correspond to those used in Papa *et al*. 2008 [52].

As reported by Papa *et al*., repeat #7 was by far the most abundant and was significantly more common in *H. erato *(detected in 4.3% of unigenes) than *H. melpomene *(2.2%; one-tailed test of proportions, p < .001). To better characterize the nature of these *Heliconius *repeats we further examined repeat #7. Instances of this repeat typically fell outside ORF predictions from the PartiGene software, suggesting that when present in transcribed sequence it occurs in 3' or 5' untranslated regions of genes, not in the coding sequence. For one *H. erato *accessory gland unigene (Her00086), three of five ESTs lacked the repeat sequence; this indicates that for at least one locus the repeat motif is polymorphic (i.e. present/absent) among individuals pooled for library construction. Finally, BLAST searches in GenBank revealed repeat #7 was present in the introns of two additional *Heliconius *species (*H. doris*, *mannose phosphate isomerase*, [GenBank:AF413748]; *H. himera*, *dopa decarboxylase I *[GenBank:AY437779]). These results are consistent with the interpretation of Papa *et al*. that these repetitive elements likely arise from the replication and insertion of transposable elements that are common among *Heliconius *butterflies.

These repeats present a practical problem when using BLAST to identify homologous unigenes within and between *Heliconius *species. Such searches may generate significant alignment scores between unigenes either because the transcribed genes are truly homologous or because a repetitive element occurs in both transcripts. We therefore used the masked unigenes for all BLAST searches between *H. erato *and *H. melpomene *libraries. We assume that significant similarity scores produced using these masked unigenes indicate homologous transcripts and not spurious similarity due to sharing of repetitive sequence.

### Comparisons of Accessory Gland and Wing Libraries

**Figure 4:**
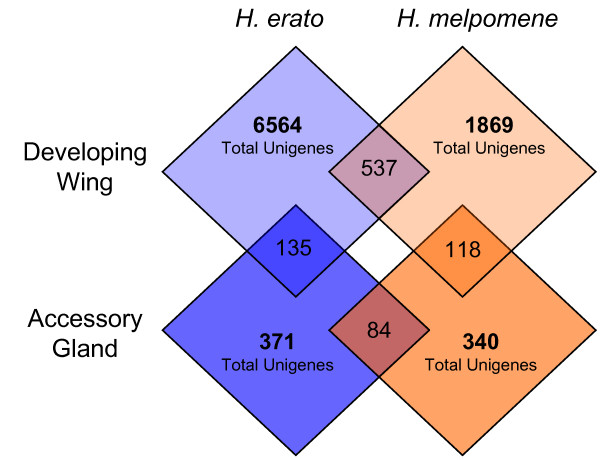
**Counts of unigenes found in common between male accessory gland and developing wing cDNA libraries**. Numbers in overlapping areas of diamonds are counts of reciprocal best BLAST hits (E-value < 10^-10^) between unigenes from each library. Numbers in non-overlapping areas of diamonds are the total number of unigenes obtained from each library.

We used the criterion of high-scoring reciprocal best BLAST hits (RBBH; E-value < 10^-10^) to explore overlaps in the transcripts sampled from accessory gland and wing libraries (Fig 4). For this analysis we assume that a highly significant RBBH between unigenes indicates that these transcripts originate from the same locus (within species) or orthologous loci (between species). However, we fully recognize that such questions of identity and orthology can only be conclusively determined in the context of complete genome sequences and that the modest number of ESTs generated for some of these libraries hardly represents an exhaustive profiling of the tissue's transcriptome. Nonetheless, contrasting the overlap in ESTs sampled between species or tissues is useful for identifying qualitative differences and similarities in the results. For instance, comparing wing and accessory gland RBBHs between species as a percentage of total *H. melpomene *unigenes yields similar results: ESTs sampled from accessory glands (84/340 = 24.7%) and from wing (537/1869 = 28.7%) show similar proportions of unigenes shared between species. We used *H. melpomene *unigenes as the denominator in this comparison because *H. melpomene *has fewer ESTs sampled from both tissues, which we assume is a limiting factor in identifying RBBHs. These results suggest consistency between libraries, but must be interpreted with caution due to differences in library construction and EST sampling. We anticipate that the numbers of genes identified in common between these species will increase dramatically as more ESTs become available.

A useful comparison can also be made between tissues within species. Genes common among accessory gland ESTs but absent from wing ESTs are promising candidates for encoding seminal fluid proteins. Encouragingly, the results from both species clearly indicated that a majority of transcripts sampled from the accessory glands were not present in wing tissue unigenes (Fig 4). In *H. erato *only 141 accessory gland unigenes had high-scoring BLAST hits (E-value < 10^-10^) to wing unigenes, of which 135 were RBBHs. In *H. melpomene *there were 125 high-scoring BLAST hits to wing unigenes, with 117 RBBHs. Therefore, in both species about 65% of accessory gland unigenes were not found among transcripts sampled from wing tissue.

Considered broadly, this lack of overlap indicates that the set of genes sampled from accessory glands is qualitatively different from the set of genes previously sampled from developing wing tissues. The wing libraries were not screened or subtracted and were well-sampled (*H. erato*, 17,573 ESTs;*H. melpomene*, 4,976 ESTs) [27], so this discrepancy in genes sampled from the two tissues likely reflects two phenomena, one biological and one methodological. Biologically, it might be that patterns of gene expression are quite different between these two tissues; the differences in sampled genes therefore may reflect substantial differences in transcript abundances. However, verifying this would require much deeper EST sampling and a methodologically consistent approach for profiling transcripts (e.g. microarrays). Apart from any underlying biological differences, our sampling method was also explicitly biased: we probed our libraries with female cDNA and sequenced non-hybridizing clones in order to enrich our ESTs for male-specific transcripts. Although we do not have unbiased samples for comparison, the relatively low and stable proportion of unigenes shared between wing and accessory gland ESTs suggests that the enrichment for male-specific transcripts was moderately successful and that the resulting accessory gland ESTs will prove a useful resource for identifying seminal fluid proteins. Nonetheless, the enrichment process was clearly not perfect. For instance, many accessory gland unigenes showed highly significant BLAST hits to well-known 'housekeeping' genes which presumably exhibit little differential expression between sexes (e.g. *cystathionine beta-synthase*,*elongation factor 1-α*, ribosomal proteins, and ribosomal RNAs) (see additional file 3).

### Highly abundant transcripts are repetitive, secreted proteins

**Figure 5:**
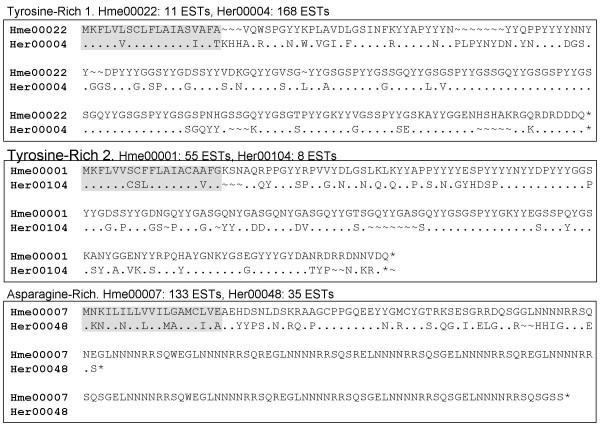
**Amino acid alignments between *H. melpomene *and *H. erato *of candidate spermatophore proteins**. These sequences are from the most abundant transcripts in each of three groups of highly abundant, repetitive proteins observed in the male accessory gland cDNA libraries. Dots (.) indicate identity between sequences; tildes (~) represent alignment gaps; filled boxes denote predicted signal sequences.

Both the *H. erato *and *H. melpomene *libraries contained a few unusually abundant transcripts (i.e. >20 ESTs per unigene, Maximum: *H. erato*, 168; *H. melpomene*, 133; Table 2). BLAST searches within libraries revealed that these abundant transcripts were highly similar to several other less-abundant transcripts. Overall there were three such groups each composed of about ten unigenes. Each group encoded highly repetitive proteins, two with a repeat structure rich in tyrosine and one rich in asparagine; all had a predicted signal peptide (Fig 5). These same three groups were identified in both species and sequences within groups were clearly similar between species. Similarities were also evident in the repeat structure present in the two tyrosine-rich groups. Unfortunately, extensive indel variation and the repetitive nature of these sequences precluded reliable alignments among any of these unigenes. Therefore robust inferences of homology between these sequences were not possible either within groups or between species. Sequences included in Figure 5 have been submitted to GenBank [GenBank: FJ465130 – FJ465135].

It is worth noting that the *H. erato *transcripts from the third (asparagine-rich) group exhibited some exceptions to the patterns uniting these groups of proteins. First, they completely lacked the repetitive asparagine-rich C-terminus motif that characterizes their *H. melpomene *counterparts (Fig 5). Nonetheless, the *H. erato *transcripts were clearly homologous to the non-repetitive N-termini of the *H. melpomene *sequences. Second, there were only two unigenes in this *H. erato *group while the other groups contained around ten unigenes. Nonetheless, one of these two unigenes, Her00048, was comprised of 35 ESTs and was the third most abundant transcript sampled from that library.

Sequences from these three groups of transcripts did exhibit weak but significant similarity to sequences or protein domains in public databases (determined via BLAST and InterProScan). However, after inspecting these results we concluded that these similarities did not reflect true homology. Rather, these significant scores arose spuriously from matches to the repetitive motifs found in these sequences. No similar sequences were found among wing unigenes.

### Accessory gland ESTs facilitate identifying reproductive proteins

In insects, most work identifying seminal fluid proteins has focused on two major criteria: enriched expression in accessory glands and the presence of a computationally predicted signal peptide [42,57]. Genes (and their encoded proteins) meeting these two criteria are commonly called ACPs (accessory gland proteins) and early work in *Drosophila *using western blots generally supported the assumption that these proteins are transferred to females in seminal fluid [44,46,58]. More recent proteomic studies have broadly confirmed this assumption but have also revealed that many genes encoding seminal fluid proteins show significant expression outside of accessory glands [43,45,47]. In light of this precedent, we focused on the highly abundant tyrosine- and asparagine-rich transcripts to demonstrate the utility of our ESTs for identifying *Heliconius *ACPs and seminal fluid proteins.

**Figure 6:**
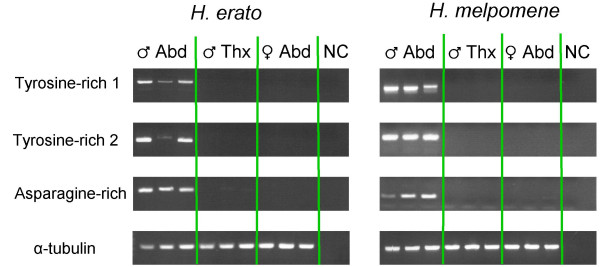
**Tissue specific patterns of expression for candidate spermatophore proteins assayed via reverse transcription PCR**. Patterns of expression for the three groups of highly abundant accessory gland transcripts and *α-tubulin *were assayed in three males (abdomen and thorax) and three females (abdomens only). PCR primers were designed to amplify universally from all transcripts in each of the three groups, two corresponding to tyrosine-rich proteins and one to asparagine-rich proteins. First-strand cDNA synthesized from equal concentrations of total RNA was used as template. For each primer set, equal amounts of PCR amplicon (ranging between 4 and 9 μL) were electrophoresed on 1.2% agarose gels. NC = negative control (no template added to PCR mix).

The high abundance of these transcripts in the accessory gland make them obvious candidates for being ACPs encoding seminal fluid proteins. The presence of a signal peptide in all groups meets one of the major criteria for identifying insect ACPs. None of these sequences were found among ESTs generated from developing wing tissue in either species; this absence, contrasted with their abundance among accessory gland ESTs, provides support for the criterion of accessory-gland biased expression. We further evaluated this criterion using reverse transcription PCR (RT-PCR) to amplify these transcripts from male and female abdomen and also male thorax. Species-specific primers were designed to fall in regions of robust alignment between all members of each group so that tests of tissue-specific patterns of expression were inclusive of all transcripts and were therefore conservative. We used primers designed for *α-tubulin *as a positive control. RT-PCR results were similar for all three groups of transcripts in both species: there was robust amplification from male abdomen but weak or no amplification from male thorax and female abdomen (Fig 6). In contrast, *α-tubulin *amplified robustly from all tissues. These three observations: 1) the presence of a predicted signal peptide, 2) the discrepancy in EST abundance between wing and accessory gland tissue, and 3) the tissue-specific patterns of expression, are consistent with these transcripts being ACP genes and suggest they encode seminal fluid proteins.

We used shotgun peptide sequencing (2d-LC/MS) to search for these candidate seminal fluid proteins in *H. erato *spermatophores, the proteinaceous packet containing sperm and seminal fluid transferred from males to females during copulation. In *Heliconius*, spermatophores can be easily and cleanly dissected from freshly mated females; we crushed the spermatophores in buffer, pelleted the remnants via centrifugation, and reserved the supernatant for analysis. Tandem mass spectra generated from this supernatant were searched against protein translations of the combined *H. erato *and *H. melpomene *accessory gland and wing unigenes. This search yielded a significant match (p < .005) to the Tyrosine-Rich 1 group of proteins from *H. erato *(Fig. 5), which includes the single most abundant transcript (168 ESTs) sampled from the accessory glands. This result confirms that at least one of the three groups of transcripts encodes a seminal fluid protein. More generally, it demonstrates the utility of these accessory gland ESTs as a resource for identifying seminal fluid proteins in *Heliconius *butterflies.

Unfortunately, due to the lack of similarity to other known sequences it is difficult to predict the molecular function of the Tyrosine-Rich 1 group of proteins or the two others which were not detected in the 2d-LC/MS experiment. However, we note the similarity between our results and other studies in crickets reporting abundant, hyper-variable, repetitive, secreted proteins with accessory gland biased expression and which were present in the spermatophore [36,41,43]. These authors speculate that the abundance and repetitive nature of these proteins suggest they are structural components of the spermatophore, which are generally known to be encoded by male insect accessory glands [41,48]. Although the tyrosine- and asparagine- rich *Heliconius *proteins do not appear to be homologous to the cricket proteins or another spermatophore protein reported in beetles [59,60], if these *Heliconius *proteins are structural components of the spermatophore it offers a possible explanation for the failure to detect the two additional groups in our proteomic assay. These proteins are unlikely to be water-soluble and the centrifugation step could have removed most of the spermatophore's structural components from the supernatant which was analyzed. Future work on the biochemical properties and structure of these three proteins will be informative in this matter, as will precisely specifying where in the spermatophore the Tyrosine-Rich 1 proteins are located. Alternately, it may be that the two undetected proteins are not present in the spermatophore and are not seminal fluid proteins, in which case useful biological insights will likely arise from investigating this functional difference between otherwise similar proteins. Either way, this combined approach using focused EST sequencing, *in silico *and *in vitro *expression assays, and proteomic analyses has successfully identified novel and noteworthy *Heliconius *proteins for future research.

## Conclusion

We report the successful sequencing of 936 ESTs, corresponding to 371 unigenes, and 1033 ESTs, corresponding to 340 unigenes, from the male accessory glands of *H. erato *and *H. melpomene*, respectively. Overall the results from the two species were very similar; our analyses did not reveal any obvious patterns that might reflect differences between the pupal and adult mating system. Approximately one-third of these unigenes showed no significant BLAST similarity to sequences in GenBank's non-redundant databases, indicating that a large proportion of novel genes are expressed in *Heliconius *male accessory glands. In both species only a third of accessory gland unigenes were also found among unigenes derived from wing tissue. About 25% of unigenes from both species encode secreted proteins. This includes three distinct groups of unigenes which consist of a few highly abundant transcripts and several less-abundant related transcripts, all differentiated by extensive indel variation. Patterns of tissue-specific expression suggest that they are ACPs; proteomic analysis confirmed the presence of proteins from one of these groups in the spermatophore.

These EST sequences lay the foundation for future research investigating the patterns and processes of molecular evolution among reproductive proteins in *Heliconius *butterflies. In particular, the striking dichotomy in mating systems offers a promising opportunity to explore the role of post-mating sexual selection in contributing to the rapid evolution of reproductive proteins. More generally, *Heliconius *butterflies are a remarkable system for investigating patterns of genetic diversity in the context of well-characterized ecological and phenotypic diversity. The two species studied here are also the focal taxa for research examining the genetic basis of wing pattern diversity in *Heliconius*. Our results comprise the first major expansion of genomic-scale research into other aspects of *Heliconius *biology. They therefore mark a significant advance in the development of these species, and the *Heliconius *genus, as model systems for connecting various aspects of genomic, phenotypic, and ecological diversity.

## Methods

### RNA isolation and cDNA library construction

Male accessory glands were dissected from 11 adult male *Heliconius erato petiverana *(from stocks maintained at the University of Puerto Rico, Rio Piedras) and 10 adult male *Heliconius melpomene rosina *(from stocks maintained at the University of Texas, Austin). Tissue samples were placed immediately in TRIZOL reagent (Invitrogen, Carlsbad, CA) and homogenized. These and other subsequent total RNA extractions were done using TRIZOL and following the manufacturer's protocol.

Two directional cDNA libraries were constructed, one for each species, using the Creator SMART cDNA library kit (Clontech BD Bioscience, Mountain View, CA). Briefly, first-strand cDNA was reverse transcribed from 1.2 μg (*H. erato*) and .7 μg (*H. melpomene*) total RNA. Second-strand synthesis and amplification of cDNA pools for library construction were accomplished via Long Distance-PCR using the following cycling program: 1 min denaturation at 95°, 20 cycles of 30 sec at 95° then 6 min at 68°, and a final extension step of 6 min at 68°. Primers provided by the manufacturer were used for these reactions.

Seventy-five μL of the PCR-amplified cDNA were cleaned with Qiaquick PCR clean-up kits (Qiagen, Valencia, CA) and digested with SfiI. Digested cDNA was electrophoresed on 1.2% TBE agarose gels and size-selected for transcripts >800 bp in length by gel extraction using a Qiaquick gel purification kit (Qiagen, Valencia, CA). The size-selected cDNA was ligated into the pDNR-LIB vector and used to transform electromax DH5α *E. coli *cells (Invitrogen, Carlsbad, CA) via electroporation with 2.5 kV/cm, 200 ohms, and 25 μF. Recombinant colonies were grown on chloramphenicol-selective LB agar medium. The *H. erato *and *H. melpomene *libraries contained 7 × 10^6 ^and 1.3 × 10^5 ^cfus respectively.

### Library screening and EST sequencing

To enrich for transcripts expressed primarily in male tissue, both libraries were screened with cDNA generated from female abdominal tissue and only non-hybridizing clones were sequenced. Aliquots were plated at low density on chloramphenicol-selective LB agar medium and grown overnight at 37°. Colony lifts were made on Hybond XL membranes (Amersham Biosciences, Piscataway, NJ). Cells were lysed and DNA was fixed to the membrane by dry-cycle autoclaving at 250° (5 min sterilize, 5 min dry) followed by baking at 80° for two hours.

The probe for screening was generated from total RNA isolated from a single female abdomen. Four μg total RNA were used in a first-strand reverse transcription and subsequent second-strand synthesis/PCR amplification following the method of Chenchik *et al*. [61]. Approximately 50 ng amplified cDNA was labeled with 32-P dCTP using the RADprime labeling kit (BioRad).

Before hybridization, membranes were soaked in 2× SSC solution and then incubated for 2 hours at 65° in hybridization buffer (0.5% BSA, 1 mM EDTA, 7% SDS, 0.5 M sodium phosphate). After 2 hours of pre-hybridization the radio-labeled female cDNA was added to the buffer and incubation continued overnight at 65°. Following hybridization, membranes were washed twice for 20 min with 1× SSC/0.5% SDS at 65°, rinsed twice with 2× SSC at room temperature, dried, and imaged with x-ray film using a 5-day exposure.

In addition to screening with female cDNA, the *H. melpomene *library was simultaneously screened for four highly abundant transcripts and the stuffer fragment from the pDNR vector. The four highly abundant transcripts were identified by random sequencing of 356 clones before any hybridization screen. PCR primers were designed to amplify a portion of these 5 templates and 5 ng of amplicon from each, purified with a Qiaquick PCR clean-up kit, were combined and labeled with P^32^-dCTP using the RADprime labeling kit (BioRad). This probe was added to the hybridization buffer at the same time as the probe generated from female cDNA. See Additional file 6 for primer details.

Clones which failed to hybridize were manually picked into 50 μL 5 mM Tris (pH 8.0) and lysed by heating at 99° for 5 min. One μL of this "boil prep" was used as template in a 10 μL PCR reaction using m13 primers (Clontech), platinum Taq polymerase (Invitrogen, Carlsbad, CA) and the following cycling program: initial denaturation of 95° (2 min), 35 cycles of 95° (50 sec) then 52° (1 min) then 72° (1 min), and a final extension of 72° (4 min). PCR amplified inserts were enzymatically cleaned with EXOSAP and single-pass sequenced from the 5' end using the ABI Prism BigDye Terminator Cycle Sequencing chemistry and a vector specific primer, SeqPrim3 (Additional file 6). Sequencing reactions were analyzed on an ABI 3730 automated sequencer.

### EST analysis

EST data analysis was automated using the PartiGene suite of bioinformatic software [55]. Raw sequences were trimmed of vector sequence, low-quality base calls, and poly-A tails (cutoff of 12 contiguous A's). Trimmed sequences >100 bp in length were clustered into putative unique gene objects (unigenes). Consensus sequences from each unigene were annotated via BLAST searches to public databases (e.g. GenBank, SwissProt). Local BLAST databases were also used for all-vs-all BLAST searches to identify related sequences within and between libraries. Unigenes from wing tissues were downloaded from ButterflyBase [27]. All BLAST searches were performed using the parallel-BLAST server hosted by the Cornell University computational biology service unit (cbsuapps.tc.cornell.edu). BLAST results were organized and analyzed using relational databases developed in Microsoft Access (Microsoft Corp., Redmond, WA). We screened the unigenes for nine *Heliconius *repetitive elements using the RepeatMasker software [52,56].

Putative open reading frames (ORFs) were identified and translated using the PartiGene suite's application prot4EST [62]. Prot4EST utilizes several different methods for ORF prediction, including a hidden Markov model (HMM) approach implemented in ESTScan, which requires a large training set of complete coding sequences [63]. At the time of analysis, a dataset of this type was not publicly available for any Lepidopteran species, so a 'simulated transcriptome' was generated for HMM training (J. Wasmuth, Personal Communication) [64,64]. First, codon usage statistics were estimated from pooled wing and accessory gland *Heliconius erato *unigenes for which coding sequences could be reliably identified via BLAST. Next, a 'simulated transcriptome' was generated by reverse-translating the *D. melanogaster *proteome using codon usage statistics estimated for *Heliconius erato*. The resulting data set was then submitted as a training set for ESTScan.

About one third of automatically predicted ORFs were manually inspected and, if necessary, edited using the Aligner (CodonCode Corp., Dedham, MA) or BioEdit [65] software packages. These unigenes received this extra attention either due to their inclusion in the set of orthologs for evolutionary analysis or their correspondance to the highly abundant tyrosine and asparagine rich proteins (see Results and Discussion for further information). For these unigenes, automated ORF predictions were replaced with manually edited versions for Gene Ontology annotations.

Where possible, Gene Ontology (GO) classifications were assigned to each protein translation based on BLASTX (E-value<10^-5^) similarity to entries in a GO-annotated database (UNIPROT). GO annotations were summarized using 'GO-Slim' terms [54]. This process was automated using the Annot8r application in the PartiGene package [55]. Secretory signal sequence peptides were predicted with the SignalP software [53,66].

### Patterns of tissue specific expression

We examined patterns of tissue-specific expression for a few unigenes of particular interest. Differences in expression were assayed via RT-PCR from three different tissues: male abdomen, male thorax, and female abdomen. PCR primers were designed within the predicted ORF of each unigene assayed (see Additional file 6 for primer details). Primers were designed with the Primer3 software [67]; see additonal file 6 for primer sequences. Total RNA was isolated from three adult male and female butterflies. A standard concentration of total RNA from each of these RNA extractions (*H. erato*, 1 μg; *H. melpomene*, 0.5 μg) was treated with DNase (Invitrogen, Carlsbad, CA) and reverse transcribed into single stranded cDNA using poly-T primers, SuperScript III Reverse Transcriptase (Invitrogen), and following the manufacturer's protocol. One μL of a 3-fold dilution of this cDNA was used as template in a 20 μL touch-down PCR with the following cycling parameters: initial denaturation of 95°C (2 min), 12 cycles of 95°C (30 sec) then 65-53°C (30 sec, decreasing one degree per cycle) then 72°C (2 min), 23 cycles of 95°C (30 sec) then 53°C (30 sec) then 72°C (2 min), and a final extension of 72°C (4 min). For each set of primers an equal amount of PCR amplicon (between 4 and 9 μL) from each of the nine templates was electrophoresed on a 1.2% agarose gel, stained with ethidium bromide and visualized under UV light.

### Spermatophore collections and proteomic analysis

*H. erato *individuals used in this experiment were taken from breeding stocks maintained at the Niagara Butterfly Conservatory, Niagara Falls, Ontario, Canada. Matings were performed in a 3 m × 3 m × 3 m screen cage inside a green house. Females recently emerged from their chrysalis were placed in the cage with several males taken from larger rearing populations. The cage was checked for coupled pairs approximately every 30 min. Coupled butterflies were placed in individual plastic boxes until they separated. Afterwards males were discarded and the spermatophore was immediately dissected out of the female's bursa copulatrix. Dissections were performed in ice-cold insect Ringer's solution. A total of 12 spermatophores were homogenized in a single microfuge tube containing 75 μL cold Phosphate Buffered Saline solution and centrifuged at 4°C for 15 minutes at 13,000 rpm. The resulting supernatant was stored at -80°C and sent to the Genome BC Proteomics Centre (University of Victoria, Canada) for two-dimensional liquid chromatography tandem mass-spectrometry (2d LC/MS) proteomic analysis. We describe this experiment only briefly here; complete details are provided in additional file 7. Initial separation of the spermatophore protein sample was performed with strong cation exchange (SCX) high performance liquid chromatography (HPLC). These SCX fractions were then analyzed on a Hybrid Quadrupole-TOF LC/MS/MS Mass Spectrometer (*QStar Pulsar I*, Applied Biosystems, Foster City, CA) with data acquired automatically using the Analyst QS 1.0 software (ABI MDS SCIEX, Concord, Canada). The resulting spectra were searched using the MASCOT 2.0 software (Matrix Science, Boston, MA) against a protein database generated from *Heliconius *unigene sequences. The protein database, created using custom Perl scripts, consisted of all ORFs ≥ 10 amino acids long from all three forward reading frames from the combined *H. erato *and *H. melpomene *accessory gland and wing unigenes. It contained approximately 180,000 protein sequences derived from *Heliconius *unigenes as well as likely contaminants: pig trypsin and human keratin.

## Abbreviations

ACP: Accessory Gland Protein; BAC: Bacterial Artificial Chromosome; EEFG: Ecological and Evolutionary Functional Genomics; EST: Expressed Sequence Tag; HPLC: High; ORF: Open Reading Frame; RBBH: Reciprocal Best BLAST Hits; RT-PCR: Reverse Transcription Polymerase Chain Reaction.

## Authors' contributions

JRW conceived of the study, designed research, performed laboratory work, completed all bioinformatic analyses, and drafted the manuscript. RGH helped to conceive of the study, participated in designing the research, and helped draft the manuscript. Both authors have read and approved the final manuscript.

## Supplementary Material

Additional File 1 ***H. erato *****accessory gland unigenes.** Complete set of 371 unigene sequences derived from ESTs generated from *H. erato *male accessory cDNA libraries. Click here for file

Additional File 2 ***H. melpomene *****accessory gland unigenes.** Complete set of 340 unigene sequences derived from ESTs generated from *H. melpomene *male accessory cDNA libraries. Click here for file

Additional File 3 **Top 5 BLAST hits for accessory gland unigenes.** This file contains the 5 best BLASTX and BLASTN hits for each unigene versus GenBank. The number of ESTs per unigene is also listed here. Click here for file

Additional File 4 **Gene ontology annotations.** This file contains the flat files produced by the Annot8r software for assigning gene ontology classifications to accessory gland unigenes. Click here for file

Additional File 5 **Results from masking unigenes for repetitive elements.** This file contains the output from RepeatMasker when used to mask the unigenes for *Heliconius *specific repetitive elements. Click here for file

Additional File 6 **PCR Primers for library screening and RT-PCR.** This file contains descriptions and sequences of primers used in screening the library and testing patterns of expression via RT-PCR. Click here for file

Additional File 7 **Protocol details for 2d-LC/MS.** This file contains the complete details for the complete 2d-LC/MS experiment outlined in the methods. Click here for file
