# Supplementary material for: EST analysis of male accessory glands from Heliconius butterflies with divergent mating systems
Source: BMC Genomics. 2008 Dec 8;9:592. doi: 10.1186/1471-2164-9-592 (PMC2621208; doi:10.1186/1471-2164-9-592)
Supplement: Additional File 7 — Protocol details for 2d-LC/MS. This file contains the complete details for the complete 2d-LC/MS experiment outlined in the methods. [file 1471-2164-9-592-S7.doc]

**University of Victoria-Genome BC Proteomics Centre**

**In Solution Digest and 2D LC-MS/MS protocol**

Upon arrival at the Proteomics Centre, all samples are issued distinct identifiers (LIMS id’s) and are stored at -20ºC until they are ready to be processed.

The protein concentration of each sample is measured using a Sigma Bicinchoninic Acid kit (part # BCA 1-kt) using bovine serum albumin as a protein standard. The proteins are precipitated by adding 9 volumes of ice cold acetone and incubating overnight at 4°C. After removal of the acetone, the protein pellet is re-suspended in 25mM Triethyl ammonium bicarbonate containing 6M urea.

Proteins are reduced with 10 mM DTT for 45 minutes at 37°C and alkylated 40mM iodoacetmaide. The reaction is then quenched with 40mM DTT. Proteins are digested overnight with Promega modified porcine trypsin at 37°C at an enzyme to protein ratio of 1:20.

***Strong Cation Exchange (SCX) HPLC***

***Set-up***

A Vision Workstation (AB, Foster City, USA) was equipped with a Polysulfoethyl A (Poly LC, Columbia, MD) 100mmX4.6mm, 5um, 300A SCX column. Buffer A was 10mM KPO_4_ (pH 2.7), 25% ACN. Buffer B was 10mM KH_2_PO_4_, 25% ACN, 0.5M KCl. The flow rate was set to 0.5ml/min.

***Procedure***

Samples were brought up to 2mL with buffer A and injected onto the column. The column was allowed to equilibrate for 20 min in buffer A before a gradient was applied; 0-35% B in 30 minutes. Fractions were collected every minute after injection. The collected fractions were then reduced in volume in a Speed-Vac (Savant Instruments, Holbrook, NY) and transferred to autosampler vials (LC Packings, Amsterdam)

## LC-MS/MS Materials and Methods

***Set-up***

LC-MS/MS analysis was performed using an integrated Famos autosampler, SwitchosII switching pump, and UltiMate micro pump (LC Packings, Amsterdam) system with an Hybrid Quadrupole-TOF LC/MS/MS Mass Spectrometer (QStar Pulsar i) equipped with a nano-electrospray ionization source (Proxeon, Odense, Denmark) and fitted with a 10μm fused-silica emitter tip (New Objective, Woburn, MA). Chromatographic separation was achieved on a 75μm x 15 cm C18 PepMap Nano LC column (3μm, 100Å, LC Packings, Amsterdam) and a 300μm x 5mm C18 PepMap guard column (5μm, 100Å, LC Packings, Amsterdam) was in place before switching inline with the analytical column and the MS. The mobile phase (solvent A) consisted of water/acetonitrile (98:2 (v/v)) with 0.05% formic acid for sample injection and equilibration on the guard column at a flow rate of 100μL/min. A linear gradient was created upon switching the trapping column inline by mixing with solvent B which consisted of acetonitrile/water (98:2 (v/v)) with 0.05% formic acid and the flow rate was reduced to 200nL/min for high resolution chromatography and introduction into the mass spectrometer.

***Procedure***

Samples were brought up to 20μL with 5% ACN and 3% FA and transferred to autosampler vials (LC Packings, Amsterdam). 10uL of sample were injected in 95% solvent A and allowed to equilibrate on the trapping column for 10 minutes to wash away any contaminants. Upon switching inline with the MS, a linear gradient from 95% to 40% solvent A developed for 40 minutes and in the following 5 minutes the composition of mobile phase was increased to 20% A before decreasing to 95% A for a 15 minute equilibration before the next sample injection. MS data was acquired automatically using Analyst QS 1.0 software Service Pack 8(ABI MDS SCIEX, Concord, Canada). An information dependent acquisition method consisting of a 1 second TOFMS survey scan of mass range 400-1200 amu and two 2.5 second product ion scans of mass range 100-1500 amu. The two most intense peaks over 20 counts, with charge state 2-5 were selected for fragmentation and a 6 amu window was used to prevent the peaks from the same isotopic cluster from being fragmented again. Once an ion was selected for MS/MS fragmentation it was put on an exclude list for 180 seconds. Curtain gas was set at 23, nitrogen was used as the collision gas and the ionization tip voltage used was 2700V. If the observed A_215_ was greater than 0.1 for any fraction collected during the SCX a 2.5 hour gradient (95-50% solvent A) was used to compensate for the higher peptide concentration in that fraction.

***Data Analysis***

Data files were processed using MASCOT (www.matrixscience.com). Carbamidomethyl cysteine was used as a fixed modification, oxidation of methionine and deamidation of asparagine and glutamine were selected as variable modifications. A maximum of one missed cleavage was allowed.
